# Elterliche Reaktionen auf kindliche Schmerzen: Die Rolle von kindlichen und elterlichen Somatisierungs- und Angstsymptomen

**DOI:** 10.1007/s00482-021-00551-8

**Published:** 2021-04-30

**Authors:** Maren K. Wallrath, Adam Geremek, Julian Rubel, Clemens Lindner, Tanja Hechler

**Affiliations:** 1 Abteilung für Klinische Psychologie und Psychotherapie des Kindes- und Jugendalters, Universität Trier, Universitätsring 15, 54286 Trier, Deutschland; 2 Klinik für Kinder- und Jugendpsychiatrie, Helios Klinikum, Schleswig, Deutschland; 3 Abteilung für Psychotherapieforschung, Justus-Liebig-Universität Gießen, Gießen, Deutschland

**Keywords:** Elternverhalten, Katastrophisieren, Zuwendung, Chronischer Schmerz, Angst, Kinder und Jugendliche, Parental behaviour, Catastrophizing, Solicitousness, Chronic pain, Pediatric and adolescent anxiety

## Abstract

**Hintergrund:**

Elterliche kognitiv-affektive und verhaltensbezogene Reaktionen können die Chronifizierung von kindlichen Schmerzen beeinflussen. Unklar ist, ob Mütter und Väter unterschiedlich reagieren und inwieweit Top-down- (elterliche Somatisierung, Angstsymptome) und Bottom-up-Variablen (kindliche schmerzbezogene Beeinträchtigung, Angstsymptome) die elterlichen Reaktionen modulieren.

**Ziele der Arbeit:**

(1) Vergleich der Somatisierung, Angstsymptome und elterlichen Reaktionen (Katastrophisieren, Zuwendung) von Müttern und Vätern chronisch schmerzkranker Kinder und (2) Untersuchung des Einflusses von Top-down- und Bottom-up-Variablen auf die elterlichen Reaktionen.

**Methode:**

Eltern-Kind-Triaden (Kind, Mutter, Vater; je *N* *=* 21, *Gesamt‑N* *=* 63; Kinder: 50 % weiblich, 11–19 Jahre, ∅15,14 Jahre) wurden während einer kinder- und jugendpsychiatrischen Behandlung ihrer chronischen Schmerzen hinsichtlich der kindlichen Schmerzen und Angstsymptome, elterlicher Somatisierung und Angstsymptome und elterlichen Reaktionen mit validierten Fragebögen erfasst.

**Ergebnisse:**

Mütter und Väter unterschieden sich nicht in Somatisierung, Angstsymptomen und Reaktionen. Eltern katastrophisierten stärker, wenn ihre Kinder sowohl unter Angstsymptomen als auch unter stärkerer schmerzbezogener Beeinträchtigung litten. Elterliche Zuwendung war verstärkt, wenn Eltern selbst Angstsymptome angaben. Jüngere Kinder und Mädchen erhielten mehr Zuwendung.

**Diskussion:**

Im Einklang mit vorherigen Studien zeigt sich, dass elterliche und kindliche Angstsymptome, nicht aber das elterliche Geschlecht als modulierende Faktoren der elterlichen maladaptiven Reaktionen eine Rolle spielen. Dies sollte in Prävention und Therapie von Kindern mit chronischen Schmerzen und deren Bezugspersonen berücksichtigt werden.

## Abstract (en)

**Background:**

Parental cognitive-affective and behavioural responses impact on the chronification of the child’s pain. Whether mothers and fathers differ in their responses and whether top-down variables (parental somatization, anxiety symptoms) and bottom-up variables (child’s pain-related disability, anxiety symptoms) impact on parental responses remains unresolved.

**Objectives:**

(1) A comparison of maternal and paternal somatization, anxiety, symptoms and their responses (parental catastrophizing, solicitousness) to children with chronic pain; (2) an analysis of the impact of top-down variables (parental somatization, anxiety symptoms) and bottom-up variables (child’s pain-related disability, anxiety symptoms) on parental maladaptive responses.

**Methods:**

Pediatric chronic pain and anxiety symptoms, parental somatization and anxiety symptoms, as well as parental responses in *N* =21 parent–child triads (child, mother, father; *N* *=* 21 each, total-*N* *=* 63; children: 50% female, 11–19 years, ∅15.14 years) were assessed via validated questionnaires during child and adolescent psychiatric treatment for child chronic pain.

**Results:**

Mothers and fathers did not differ in somatization, anxiety symptoms and responses. Parental catastrophizing was higher if the child suffered from anxiety symptoms and from pain-related disability. Parental solicitousness was higher if parents reported more own anxiety symptoms. Younger children and girls received more solicitous responses.

**Conclusion:**

As shown by previous studies, parental and child anxiety symptoms, but not parental gender, play a pivotal role in modulating parental maladaptive pain-related responses. This should be taken into account in prevention as well as in the treatment of children with chronic pain and their caregivers.

Kindliche chronische (primäre) Schmerzen hängen neben der kindlichen, Vulnerabilität auch von den elterlichen schmerzbezogenen Reaktionen ab [28]. Nach dem Empathiemodell von Goubert et al. [11] spielen Top-down-Variablen (elterlich) und Bottom-up-Variablen (kindbezogen) hinsichtlich der maladaptiven elterlichen Reaktionen (Katastrophisieren, Zuwendung) eine zentrale Rolle. Erkenntnisse bezüglich modulierender Faktoren können helfen, Risikogruppen von Eltern zu identifizieren und präventive und therapeutische Angebote zu entwickeln, um der Chronifizierung von Schmerzen bei Kindern vorzubeugen.

## Hintergrund und Fragestellungen

Chronische Schmerzen im Kindes- und Jugendalter sind mit einer weltweiten Prävalenz von ca. 44 % weit verbreitet [10] und stellen die Kinder und ihre Bezugspersonen vor substanzielle Herausforderungen. Neben schmerzbezogenen Einschränkungen der Kinder im Alltag und der hohen Komorbidität mit anderen psychischen Störungen [20, 29] zeigen auch ihre Eltern ein erhöhtes Stresslevel, insbesondere wenn ihre Kinder in vollstationärer Behandlung sind [18].

Eigene chronische Schmerzen bei Eltern gelten nach dem Modell der familialen Transmission von chronischen Schmerzen von Stone und Wilson [28] als Risikofaktor für die Chronifizierung von kindlichem Schmerz [5, 28]. Betroffene Eltern katastrophisieren stärker bezüglich des kindlichen Schmerzes und zeigen mehr zuwendendes Verhalten [31]. Auch Eltern mit Angstsymptomen zeigen verstärktes schmerzbezogenes Katastrophisieren und vermehrte zuwendende Reaktionen [30]. Maladaptive elterliche kognitiv-affektive und/oder verhaltensbezogene Reaktionen [3] scheinen demnach bei der Chronifizierung der kindlichen Schmerzen eine bedeutende Rolle zu spielen [5, 13]. Dabei ist nach wie vor unklar, welche Prozesse das Ausmaß der maladaptiven elterlichen Reaktionen beeinflussen. Basierend auf dem Empathiemodell von Goubert et al. [11] können dabei Top-down- (hier: elterliche Variablen wie die elterliche Somatisierung und Angstsymptome) und Bottom-up-Prozesse (hier: kindliche Variablen wie die schmerzbezogene Beeinträchtigung und Angstsymptome) angenommen werden. Das Konstrukt der Somatisierung beschreibt die Wahrnehmung körperlicher Dysfunktionen [7], die als belastend empfunden werden und in deutlicher Weise die Funktionalität im Alltag einschränken sowie ausgeprägte und unverhältnismäßige Gedanken, Gefühle und Verhaltensweisen bezüglich der körperlichen Symptome auslösen [1]. Als schmerzbezogene Beeinträchtigung gelten Auswirkungen des chronischen Schmerzes auf die Schulfehltage, verringerte Sozialkontakte und Freizeitaktivitäten sowie die Vernachlässigung von Pflichten [15]. In einer der ersten Studien, die beide Prozesse – „top-down“ und „bottom-up“ – einbezogen haben, konnte in einer Stichprobe aus *N* *=* 118 Eltern mit chronischen Schmerzen und Angstsymptomen gezeigt werden, dass es vorwiegend Top-down-Prozesse wie die elterliche Angst waren, die das Ausmaß des elterlichen Katastrophisierens modulierten [30]. Neben dem Mangel an Studien, die Top-down- und Bottom-up-Prozesse untersucht haben, fokussieren die meisten Studien nach wie vor vorwiegend auf Mütter. Zwar gibt es in den wenigen existierenden Studien mit beiden Elternteilen widersprüchliche Ergebnisse hinsichtlich der Unterschiede von Müttern und Vätern im schmerzbezogenen Elternverhalten [8, 30], jedoch zeigt sich in der Geschlechterforschung [32] sowie in vergleichbaren Samples [13] verstärktes Katastrophisieren bei Frauen.

Die vorliegende Studie verfolgt daher folgende Ziele:

Vergleich von Müttern und Vätern chronisch schmerzkranker Kinder hinsichtlich der Top-down-Variablen (Somatisierung und Angstsymptome) sowie der maladaptiven, schmerzbezogenen elterlichen Reaktionen auf kindlichen Schmerz (Katastrophisieren, Zuwendung), wobei verstärktes Katastrophisieren wie auch stärkere Zuwendung bei Müttern im Vergleich zu Vätern erwartet wird [13, 32].

Untersuchung des Einflusses von Top-down-Variablen (elterliche Somatisierung und Angstsymptome) und Bottom-up-Variablen (kindliche schmerzbezogene Beeinträchtigung und Angstsymptome) auf maladaptive schmerzbezogene elterliche Reaktionen (Katastrophisieren, Zuwendung). Hierbei werden mehr maladaptive Reaktionen von Eltern erwartet, die eine verstärkte Somatisierung und Angstsymptome zeigen [5, 28, 30, 31]. Außerdem wird ein positiver Zusammenhang zwischen kindlicher schmerzbezogener Beeinträchtigung und kindlichen Angstsymptomen mit elterlichen maladaptiven Reaktionen erwartet [5]. Bei der Analyse der Kovariaten Alter und Geschlecht wird vermutet, dass jüngere Kinder und Mädchen mehr Zuwendung erhalten [23].

## Studiendesign und Untersuchungsmethoden

### Stichprobe

Zwischen 04/2016 und 03/2018 wurden in der Kinder- und Jugendpsychiatrie der Helios Klinik Schleswig *N* *=* 21 Mütter, *N* *=* 21 Väter und *N* *=* 21 chronisch schmerzkranke Kinder (∅15,14 Jahre alt [Standardabweichung, sd = 2,00, Range: 11–19], 50 % weiblich) befragt (Total-*N* *=* 63). Die Kinder waren aufgrund einer chronischen Schmerzstörung nach ICD-10 ambulant (*N* *=* 6) oder stationär (*N* *=* 15) in Behandlung. Die ambulant behandelten Kinder waren im Schnitt jünger als die stationär behandelten (Alter ambulant: 12,83 [sd = 1,47], Alter stationär: 16,07 [sd = 1,34], *t* (19) = −4,879, *p* =0,000), sonst zeigten sich keine Unterschiede.

Die demografischen Daten von Eltern und Kindern sind Tab. 1 und 2 zu entnehmen.

**Table 1:** 

			Kinder (*N* *=* 21)
Alter (M [sd, Range])			15,14 (2,00, 11–19)
Geschlecht			*N* *=* 11 (55,0 %) männlich *N* *=* 10 (45,0 %) weiblich
Lebensmittelpunkt	Beide Elternteile		90,4 % (*N* *=* 19)
	Mutter		4,8 % (*N* *=* 1)
	Vater		4,8 % (*N* *=* 1)
Sorgerecht	Beide Elternteile		90,4 % (*N* *=* 19)
	Mutter		9,5 % (*N* *=* 2)
Schulform	Realschule		9,5 % (*N* *=* 2)
	Gesamtschule		47,6 % (*N* *=* 10)
	Gymnasium		33,3 % (*N* *=* 7)
	Andere		9,6 % (*N* *=* 2)
Primäre Diagnose	Chron. Schmerzst. mit som. und psych. Fakt. (F45.41)		95,2 % (*N* *=* 20)
	Anhaltende somatoforme Schmerzst. (F45.40)		4,8 % (*N* *=* 1)
Sekundäre Diagnose	Sonstige emot. Störungen des Kindesalters (F93.8)		52,4 % (*N* *=* 11)
	Asperger-Syndrom (F84.5)		4,8 % (*N* *=* 1)
	Soziale Phobie (F40.1)		4,8 % (*N* *=* 1)
	Zwangsgedanken und -handlungen, gemischt (F42.2)		9,5 % (*N* *=* 2)
	Leichte depressive Episode (F32.0)		4,8 % (*N* *=* 1)
	Mittelgradige depressive Episode (F32.1)		9,5 % (*N* *=* 2)
	Agoraphobie mit Panikstörung (F40.01)		4,8 % (*N* *=* 1)
	Nichtorganische Insomnie (F510)		4,8 % (*N* *=* 1)
Angstsymptome (DISYPS-ANZ) auffällig	Trennungsangst		9,5 % (*N* = 2)
	Generalisierte Angst		66,7 % (*N* = 14)
	Soziale Phobie		61,9 % (*N* = 13)
	Spezifische Phobie		57,1 % (*N* = 12)
	Gesamt		81,0 % (*N* = 17)
Schmerzort *(Mehrfachnennungen möglich)*	Kopfschmerz		76,2 % (*N* *=* 16)
	Migräne		38,1 % (*N* *=* 8)
	Bauchschmerz		4,8 % (*N* *=* 1)
	Gelenkschmerz		23,8 % (*N* *=* 5)
	Rückenschmerz		–
Schmerzstärke (0–10)	Aktuell	Kindsurteil	6,24 (2,34) [0–9]
		Elternurteil	6,13 (1,96) [0–9]
	Letzte 7 Tage	Kindsurteil	8,30 (1,30) [5–10]
		Elternurteil	7,50 (1,43) [3–9]
	Schwächste Schmerzen letzte 7 Tage	Kindsurteil	2,25 (1,89) [0–6]
		Elternurteil	2,55 (2,25) [0–9]
	Meistens	Kindsurteil	5,43 (2,04) [0–8]
		Elternurteil	4,89 (1,67) [1–8]
Schmerzbezogene Beeinträchtigung	PPDI (M [sd]) Range: 12–60	Kindsurteil	37,57 (5,82) [27–47]
		Elternurteil	39,50 (6,74) [28–52]

*DIKJ* Depressionsinventar für Kinder und Jugendliche [27],* DISYPS-ANZ* Angstskala des Diagnostik-Systems für psychische Störungen nach ICD-10 und DSM-IV für Kinder und Jugendliche – II [10]*, PPDI* Pediatric Pain Disability Index [20], *M* Mittelwert, *sd* Standardabweichung

**Table 2:** 

		Väter (*N* *=* 21)	Mütter (*N* *=* 21)	Vergleich Väter vs. Mütter
Alter (M [sd])		50,76 (7,36)	46,05 (4,31)	–
Anzahl Kinder (M [sd, Range])		1,95 (0,97, 1–4)	1,85 (0,81, 1–4)	–
Staatsangehörigkeit deutsch		95,2 %	85,7 %	–
Beziehungsstatus	Ledig	–	4,8 % (*N* *=* 1)	–
	Verheiratet	90,5 % (*N* *=* 19)	85,7 % (*N* *=* 18)	–
	Getrennt lebend	4,8 % (*N* *=* 1)	–	–
	Verwitwet	–	4,8 % (*N* *=* 1)	–
	Neue Partnerschaft	4,8 % (*N* *=* 1)	4,8 % (*N* *=* 1)	–
Bildungsabschluss	Hauptschule	4,8 % (*N* *=* 1)	4,8 % (*N* *=* 1)	–
	Realschule	19,0 % (*N* *=* 4)	19,0 % (*N* *=* 4)	–
	Abitur	19,0 % (*N* *=* 4)	19,0 % (*N* *=* 4)	–
	Ausbildung/Lehre	23,8 % (*N* *=* 5)	33,3 % (*N* *=* 7)	–
	Fachhochschule/Universität	33,3 % (*N* *=* 7)	23,8 % (*N* *=* 5)	–
Berufstätigkeit	Vollzeit	81,0 % (*N* *=* 17)	14,3 % (*N* *=* 3)	–
	Teilzeit	–	57,1 % (*N* *=* 12)	–
	Schichtarbeit	9,5 % (*N* *=* 2)	–	–
	Nicht berufstätig	9,5 % (*N* *=* 2)	19,0 % (*N* *=* 4)	–
	Andere	–	9,5 % (*N* *=* 2)	–
Elterliche Angstsymptome (DASS) *(M [sd])* *Über Cut-off (>* *6)*		1,10 (1,80)	1,58 (2,36)	t (37) = −0,714
		4,8 % (*N* *=* 1)	4,8 % (*N* = 1)	*p* =0,480
Elterliche Somatisierung (SCL) *(M [sd])* *Auffälliger T‑Wert (>* *0,92)*		0,31 (0,32)	0,55 (0,50)	*t* (38) = −1,829
		9,6 % (*N* *=* 2)	19,2 % (*N* *=* 4)	*p* =0,075
SCL Gesamtbelastung (GSI)* (M [sd])* *Auffälliger T‑Wert (≥* *0,68 bzw. ≥* *0,71)*		0,30 (0,35)	0,46 (0,53)	*t* (40) = −1,209
		14,3 % (*N* *=* 3)	14,3 % (*N* *=* 3)	*p* =0,234
ISEV‑E Zuwendung *(M [sd])*		3,18 (0,75)	3,17 (0,69)	*t* (39) = 0,074 *p* =0,941
PCS‑P Katastrophisieren *(M [sd])*		23,48 (9,39)	25,24 (9,80)	*t* (40) = −0,595 *p* =0,555

*DASS* Depressions-Angst-Stress-Skala [28],* SCL* Symptom-Checkliste [11],* ISEV‑E* Inventar zum schmerzbezogenen Elternverhalten [19], *PCS‑P* Pain Catastrophizing Scale for Parents [18], *M* Mittelwert, *sd* Standardabweichung

### Prozedere

Die Studie wurde von der Ethikkommission der Ärztekammer Schleswig-Holstein bewilligt (AZ 057/11, II).

Die Eltern und ihre Kinder wurden beim Erstkontakt in der Ambulanz der Kinder- und Jugendpsychiatrie bei vorhandenem Einverständnis in die Studie eingeschlossen und füllten die Fragebögen zeitnah bei sich zu Hause aus. Alle Beteiligten wurden darauf hingewiesen, die Fragebögen allein und für sich auszufüllen. Das Ausfüllen der Fragebögen nahm etwa 30–60 min in Anspruch.

### Messinstrumente

#### Schmerzsymptome der Kinder – Kindsangaben

Zur Erhebung des Schmerzorts, der -häufigkeit und der -intensität des kindlichen Schmerzes wurde der *Deutsche Schmerzfragebogen für Kinder und Jugendliche (DSF-KJ; *[25]*)* genutzt.

#### Schmerzbezogene Beeinträchtigung des Kindes – Kindsangaben

Die schmerzbezogene Beeinträchtigung wurde mit dem *Pediatric Pain Disability Index (P-PDI; *[15]*)* erfasst. Die interne Konsistenz des Fragebogens liegt in der vorliegenden Studie mit α = 0,703 in einem akzeptablen Bereich.

#### Angstsymptome der Kinder – Kindsangaben

Mit dem *Diagnostik-System für psychische Störungen nach ICD-*10 *und DSM-IV für Kinder und Jugendliche zur Erfassung von Angst- und Zwangsstörungen *(*DISYPS-ANZ; *[6]*)* wurden die Angstsymptome der Kinder im Selbsturteil erfasst. Für die vorliegende Studie wurde die Gesamtskala genutzt, deren interne Konsistenz mit α = 0,886 in einem guten Bereich liegt.

#### Somatisierung der Eltern – Elternangaben

Zur Erfassung der Somatisierung der Eltern wurde die *Symptom-Checkliste (SCL-90-R*; [7]*)* genutzt. In der vorliegenden Studie wurde die Skala Somatisierung genutzt, deren interne Konsistenz mit α = 0,794 in einem akzeptablen Bereich liegt.

#### Angstsymptome der Eltern – Elternangaben

Die elterlichen Angstsymptome wurden mit der *Depressions-Angst-Stress-Skala (DASS; *[22]*)* erfasst. Die Validität des Fragebogens wurde durch Nilges und Essau [22] bestätigt. In der vorliegenden Studie wurde die Skala Angst genutzt, deren interne Konsistenz mit α = 0,716 in einem akzeptablen Bereich liegt.

#### Elterliche kognitiv-affektive Reaktionen (Katastrophisieren) – Elternangaben

Das elterliche Katastrophisieren wurde mit der deutschen Version der *Pain Catastrophizing Scale for Parents (PCS‑P; *[13]*)* erfasst. In der vorliegenden Studie wurde der Gesamtwert des Katastrophisierens verwendet, dessen Range zwischen 0 und 52 Punkten liegt. Die interne Konsistenz des Fragebogens liegt bei α = 0,904 und damit in einem exzellenten Bereich.

#### Elterliche verhaltensbezogene Reaktionen (Zuwendung) – Elternangaben

Die elterlichen verhaltensbezogenen Reaktionen wurden mit dem *Inventar zum schmerzbezogenen Elternverhalten (ISEV‑E; *[14]*)* erfasst. In der vorliegenden Studie wurde die Skala Zuwendung genutzt, deren interne Konsistenz mit α = 0,770 in einem akzeptablen Bereich liegt. Die Range des Skalenmittelwerts liegt zwischen 1 und 5 Punkten.

### Statistische Auswertung

#### Umgang mit fehlenden Werten

Die Anzahl fehlender Werte ist gering (max. 3 fehlende Werte/Variable). Es fehlte folgende Anzahl an Werten: Zuwendung 1 (2,38 %), elterliche Somatisierung 2 (4,76 %), elterliche Angstsymptome 3 (7,14 %), Alter der Eltern 1 (2,38 %). Für die Imputation fehlender Werte wurde das R‑Paket missForest [26] mit einer Maximalanzahl an 10 Wiederholungen und 100 Regressionsbäumen genutzt. Die Fit-Indizes der in diesem Datensatz vorgenommenen Imputationen wurden zur besseren Vergleichbarkeit normalisiert und zeigten sehr geringe Fehlerraten („root mean square error“ [NRMSE] = 0,14, Anteil fehlklassifizierter kategorialer Imputationen [„proportion of falsely classified“; PFC] = 0,09).

#### Analyse der Daten

Die Datenanalyse erfolgte mit SPSS [16] und R [24]. Die Daten wurden vorab auf Normalverteilung und Ausreißer geprüft. Der Vergleich der elterlichen Somatisierung und Angstsymptome sowie der schmerzbezogenen Reaktionen zwischen Müttern und Vätern erfolgte mithilfe einer ANOVA. Die Analyse der Zusammenhänge zwischen Top-down- (elterliche Somatisierung, Angstsymptome) und Bottom-up-Variablen (kindliche schmerzbezogene Beeinträchtigung, Angstsymptome) und den elterlichen schmerzbezogenen Reaktionen (Katastrophisieren, Zuwendung) wurde aufgrund der verschachtelten Datenstruktur mithilfe von Multilevelanalysen (HLM) berechnet. Für jede abhängige Variable (elterliches Katastrophisieren; elterliche Zuwendung) wurden schrittweise Modelle berechnet: (1) Berechnung des Nullmodells ohne Prädiktoren, das die Gesamtvarianz der abhängigen Variable in Variationen zwischen den Eltern und Variationen zwischen den Kindern (also innerhalb der Eltern) angibt. (2) Einbezug der Kovariaten (Alter und Geschlecht Kind und Eltern) als Prädiktorvariablen (Kovariatenmodell). (3) Einbezug der Bottom-up-Variablen (kindliche schmerzbezogene Beeinträchtigung und Angstsymptome) sowie (4) Einbezug von deren Interaktion (Modell a und b). (5) Einbezug der Top-down-Variablen (elterliche Somatisierung und Angstsymptome) sowie (6) Einbezug von deren Interaktion (Modell c und d). Für die Interaktionsmodelle (Modelle b und d) wurden alle Prädiktoren zentriert im Modell aufgenommen.

Für alle Berechnungen wurde eine Sicherheitswahrscheinlichkeit von 95 % zugrunde gelegt.

## Ergebnisse

### Charakterisierung der Stichprobe

Die Charakteristika der 63 teilnehmenden Kinder, Mütter und Väter sind in Tab. 1 und 2 dargestellt. Die Kinder waren im Schnitt 15,14 Jahre alt (sd = 2,00, Range: 11–19) und knapp zur Hälfte weiblich. Als primäre Diagnose zeigten die Kinder in 95,2 % (*N* *=* 20) eine chronische Schmerzstörung mit somatischen und psychischen Faktoren (ICD-10: F45.41). Kinder und Eltern beurteilten die durchschnittliche aktuelle Schmerzintensität sowie die schmerzbezogene Beeinträchtigung nicht signifikant unterschiedlich (Schmerzintensität: MK = 6,24 [sd = 2,34], ME = 6,13 [sd = 1,96]; *t (*37) = −0,066, *p* = 0,948; Beeinträchtigung: MK = 37,57 [sd = 5,82], ME = 39,50 [sd = 6,74]; *t (*61) = 1,119, *p* =0,27). Klinisch auffällige Angstsymptome berichteten 81,0 % (*N* *=* 17) der Kinder.

Die Charakteristika der Väter und Mütter sind in Tab. 2 dargestellt, weitere Angaben zu psychischen Symptomen finden sich in Tab. 3. Bezüglich der elterlichen Somatisierung und Angstsymptome zeigte nur ein geringer Prozentsatz der Mütter und Väter klinisch auffällige Symptome (Somatisierung: Väter: 9,6 % (*N* = 2); Mütter: 19,2 % (*N* *=* 4); Angst: je 4,8 % bzw. *N* *=* 1).

**Table 3:** 

	Väter (*N* = 21)	Mütter (*N* = 21)
**DASS Angst** *(M [sd])*	1,10 (1,80)	1,58 (2,36)
*Über Cut-off (>* *6)*	*N* *=* 1 (4,8 %)	*N* *=* 1 (4,8 %)
**DASS Depression** *(M [sd])*	4,40 (3,89)	4,16 (4,21)
*Über Cut-off (>* *10)*	*N* *=* 3 (14,4 %)	*N* *=* 2 (9,6 %)
**DASS Stress** *(M [sd])*	4,80 (3,55)	7,15 (4,89)
*Über Cut-off (>* *10)*	*N* *=* 2 (9,6 %)	*N* *=* 4 (19,2 %)
**SCL Somatisierung** *(M [sd])*	0,31 (0,32)	0,55 (0,50)
*Auffälliger T‑Wert (>* *0,92)*	*N* *=* 2 (9,6 %)	*N* *=* 4 (19,2 %)
**SCL Zwanghaftigkeit** *(M [sd])*	0,46 (0,54)	0,56 (0,68)
*Auffälliger T‑Wert (>* *0,90)*	*N* *=* 3 (14,4 %)	*N* *=* 3 (14,4 %)
**SCL Depressivität** *(M [sd])*	0,46 (0,56)	0,71 (0,77)
*Auffälliger T‑Wert (>* *0,85)*	*N* *=* 3 (14,4 %)	*N* *=* 5 (24,0 %)
**SCL Ängstlichkeit** *(M [sd])*	0,14 (0,27)	0,37 (0,59)
*Auffälliger T‑Wert (>* *0,70)*	*N* *=* 3 (14,4 %)	*N* *=* 3 (14,4 %)
**SCL Aggressivität** *(M [sd])*	0,32 (0,40)	0,53 (0,63)
*Auffälliger T‑Wert (>* *0,83)*	*N* *=* 4 (19,2 %)	*N* *=* 6 (28,8 %)
**SCL phobische Angst** *(M [sd])*	0,09 (0,23)	0,11 (0,20)
*Auffälliger T‑Wert (>* *0,57)*	*N* *=* 1 (4,8 %)	*N* *=* 1 (4,8 %)
**SCL paranoides Denken** *(M [sd])*	0,25 (0,34)	0,39 (0,70)
*Auffälliger T‑Wert (>* *1,00)*	*N* *=* 1 (4,8 %)	*N* *=* 2 (9,6 %)
**SCL Psychotizismus** *(M [sd])*	0,12 (0,24)	0,18 (0,39) *N* *=* 1 (4,8 %)
*Auffälliger T‑Wert (>* *0,50)*	*N* *=* 2 (9,6 %)	

*SCL-90®* Symptom-Checkliste [11], *DASS* Depressions-Angst-Stress-Skala [28]

### Vergleich der elterlichen Somatisierung und Angstsymptome sowie der schmerzbezogenen Reaktionen zwischen Vätern und Müttern

Entgegen der Hypothesen zeigten sich sowohl hinsichtlich der Somatisierung und der Angstsymptome als auch hinsichtlich der maladaptiven schmerzbezogenen elterlichen Reaktionen (Katastrophisieren, Zuwendung) keine signifikanten Unterschiede zwischen Vätern und Müttern (*p* > 0*,*05; Tab. 2).

### Vorhersage des elterlichen Katastrophisierens

Der Mittelwert des elterlichen Katastrophisierens lag im vorliegenden Sample bei den Vätern bei 23,48 (sd = 9,39, Range: 10–40) und bei den Müttern bei 25,24 (sd = 9,80, Range: 12–44). Die Ergebnisse des HLM finden sich in Tab. 4.

**Table 4:** 

Modell	Nullmodell	Kovariatenmodell	Modell 1a	Modell 1b	Modell 1c	Modell 1d
**Feste Effekte**						
„Intercept“	24,36^***^	46,53^**^	45,81^*^	3,21	40,67^*^	0,99
*Kovariaten*						
Alter Kind	–	−0,78	−0,97	−0,54	−0,87	−0,69
Geschlecht Kind	–	−1,34	−3,17	−2,83	0,02	−0,44
Alter Eltern	–	−0,21	−0,17	−0,18	−0,13	0,01
Geschlecht Eltern	–	0,87	1,07	0,74	−0,21	0,19
*Kindliche Variablen („bottom-up“)*						
Schmerzbezogene Beeinträchtigung (PPDI)	–	–	−0,01	−0,09	–	–
Angstsymptome (DISYPS-ANZ)	–	–	4,61	−1,10	–	–
*Interaktion* Schmerzbezogene Beeinträchtigung *×* Angstsymptome	–	–	**–**	1,56^*^	–	–
*Elterliche Variablen („top-down“)*						
Somatisierung (SCL-90®)	–	–	–	–	2,59	5,03
Angstsymptome (DASS)	–	–	–	–	1,58	1,78
*Interaktion* Somatisierung *×* Angstsymptome	–	–	–	–	–	−0,98
**Zufallseffekte**						
Level 2	29,44	16,78	16,88	5,66	7,94	18,23
Level 1	59,07	63,68	62,44	64,94	56,77	47,58

*N* *=* *21 Kinder, 42 Elternteile*. Signifikante Werte sind fett gedruckt

*PCS‑P* Pain Catastrophizing Scale for Parents [18],* SCL-90®* Symptom-Checkliste [11],* DASS* Depressions-Angst-Stress-Skala [28],* PPDI* Pediatric Pain Disability Index [20],* DISYPS-ANZ* Diagnostik-System für psychische Störungen nach ICD-10 und DSM-IV für Kinder und Jugendliche zur Erfassung von Angst- und Zwangsstörungen [10]

^***^p *=* 0, ^**^p *<* 0,001, ^*^p *<* *0,05*

Das Nullmodell zeigt, dass 76,74 % der Gesamtvarianz im elterlichen Katastrophisieren auf die Unterschiede zwischen den Elternteilen zurückzuführen sind, nur 23,26 % sind durch Unterschiede zwischen den Kindern erklärbar.

Im Kovariatenmodell zeigten sich durch Einbezug der zu kontrollierenden Variablen (Alter und Geschlecht Kind und Elternteil) keine signifikanten Einflüsse auf das Katastrophisieren der Eltern. Bei Einbezug der kindlichen schmerzbezogenen Beeinträchtigung und Angstsymptome ohne Interaktionseffekt (Modell 1a) zeigten sich ebenfalls keine signifikanten Ergebnisse. Die Interaktion zwischen kindlicher schmerzbezogener Beeinträchtigung und kindlichen Angstsymptomen (Modell 1b) zeigte einen signifikanten Effekt: Eltern von Kindern mit schmerzbezogener Beeinträchtigung katastrophisieren stärker, je mehr die Kinder auch unter Angstsymptomen leiden bzw. Eltern von Kindern mit Angstsymptomen katastrophisieren stärker, je stärker die kindliche schmerzbezogene Beeinträchtigung ist. Bezieht man beide Top-down-Variablen (Somatisierung und Angstsymptome der Eltern) ohne Interaktion mit ein (Modell 1c), zeigen sich wie auch bei Einbezug der Interaktion (Modell 1d) keine signifikanten Effekte auf das elterliche Katastrophisieren.

### Vorhersage der elterlichen Zuwendung

Der Mittelwert der elterlichen Zuwendung lag im vorliegenden Sample bei Vätern bei 3,18 (sd = 0,75, Range: 1,33–4,50) und bei Müttern bei 3,17 (sd = 0.69, Range: 2,33–4,50). Die Ergebnisse des HLM finden sich in Tab. 5.

**Table 5:** 

Modell	Nullmodell	Kovariatenmodell	Modell 2a	Modell 2b	Modell 2c	Modell 2d
**Feste Effekte**						
„Intercept“	3,17^***^	6,31^***^	7,20^***^	−0,16	5,84^***^	−0,42
*Kovariaten*						
Alter Kind	–	−0,11^*^	−0,12^*^	−0,11	−0,12^*^	−0,12^*^
Geschlecht Kind	–	0,37	0,25	0,25	0,44^*^	0,44^*^
Alter Eltern	–	−0,03	−0,03^*^	−0,03	−0,02	−0,03
Geschlecht Eltern	–	−0,15	−0,15	−0,15	−0,17	−0,17
*Kindliche Variablen („bottom-up“)*						
Schmerzbezogene Beeinträchtigung (PPDI)	–	–	−0,02	−0,02	–	–
Angstsymptome (DISYPS-ANZ)	–	–	0,27	0,16	–	–
*Interaktion* schmerzbezogene Beeinträchtigung *×* Angstsymptome	–	–	–	0,03	–	–
*Elterliche Variablen („top-down“)*						
Somatisierung (SCL-90®)	–	–	–	–	−0,09	−0,12
Angstsymptome (DASS)	–	–	–	–	0,15^*^	0,15
*Interaktion* Somatisierung *×* Angstsymptome	–	–	–	–	–	0,01
**Zufallseffekte**						
Level 2	0,10	0,00	0,00	0,00	0,00	0,00
Level 1	0,39	0,40	0,37	0,37	0,32	0,32

*N* *=* *21 Kinder, 42 Elternteile*. Signifikante Werte sind fett gedruckt

*ISEV‑E* Inventar zum schmerzbezogenen Elternverhalten Elternversion [19],* SCL-90®* Symptom-Checkliste [11],* DASS* Depressions-Angst-Stress-Skala [28],* PPDI* Pediatric Pain Disability Index [20],* DISYPS-ANZ* Diagnostik-System für psychische Störungen nach ICD-10 und DSM-IV für Kinder und Jugendliche zur Erfassung von Angst- und Zwangsstörungen [10]

^***^p *=* 0, ^**^p *<* 0,001, ^*^p *<* *0,05*

Das Nullmodell zeigt, dass 79,59 % der Gesamtvarianz in der elterlichen Zuwendung auf die Unterschiede zwischen den Elternteilen zurückzuführen sind, nur 20,41 % sind durch Unterschiede zwischen den Kindern erklärbar.

Im Kovariatenmodell wurden die zu kontrollierenden Variablen (Alter und Geschlecht Kind und Elternteil) einbezogen. Jüngere Kinder und Mädchen scheinen mehr Zuwendung zu erhalten. Ein einzelnes Modell (Modell 2a) weist darauf hin, dass ältere Eltern weniger Zuwendung zeigen als jüngere Eltern.

Der Einbezug der kindlichen schmerzbezogenen Beeinträchtigung und der kindlichen Angstsymptome ohne Interaktion (Modell 2a) sowie auch der Einbezug der Interaktion (Modell 2b) zeigten keine signifikanten Ergebnisse. Bezieht man sowohl die elterliche Somatisierung als auch die elterliche Angstsymptomatik ohne Interaktion mit ein, zeigt sich ein positiver Zusammenhang zwischen elterlichen Angstsymptomen und Zuwendung (Modell 2c): Ängstlichere Eltern reagierten mit mehr Zuwendung auf die kindlichen Schmerzen. Im Modell mit Einbezug der Interaktion zwischen elterlicher Somatisierung und Angstsymptomen (Modell 2d) zeigten sich keine signifikanten Effekte.

## Diskussion

Beim Vergleich von Vätern und Müttern zeigten sich entgegen der Hypothese eine ähnliche Ausprägung von Somatisierung und Angstsymptomen sowie ein ähnliches Ausmaß an maladaptiven elterlichen Reaktionen auf kindlichen Schmerz bei Vätern und Müttern.

Die Untersuchung des Einflusses von Top-down-Variablen (elterliche Somatisierung und Angstsymptome) und Bottom-up-Variablen (kindliche schmerzbezogene Beeinträchtigung und Angstsymptome) auf die maladaptiven elterlichen Reaktionen zeigte eine teilweise Bestätigung der Hypothesen. Eltern von Kindern mit schmerzbezogener Beeinträchtigung katastrophisierten stärker, je stärker die Angstsymptomatik der Kinder ausgeprägt war bzw. Eltern von Kindern mit Angstsymptomen katastrophisierten stärker, je höher die kindliche schmerzbezogene Beeinträchtigung war. Elterliche Variablen (Somatisierung, Angstsymptome) zeigten keinen Einfluss auf das elterliche Katastrophisieren. Die elterliche Zuwendung war entsprechend zu Vorbefunden [23] stärker bei jüngeren Kindern und Mädchen. Erwartungskonform wurde ein positiver Zusammenhang zwischen elterlichen Angstsymptomen und elterlicher Zuwendung gefunden. Weder die elterliche Somatisierung noch die Bottom-up-Variablen (kindliche schmerzbezogene Beeinträchtigung und Angstsymptome) nahmen einen Einfluss auf das Ausmaß der elterlichen Zuwendung. Im Vergleich zu vorherigen klinischen Studien an schmerzkranken Kindern [13] sowie schmerz- und/oder angsterkrankten Eltern [30] berichten die Eltern in der vorliegenden Studie über eine ähnlich hoch ausgeprägte Katastrophisierungsneigung, höher als die von Eltern von gesunden Kindern [9]. Bezüglich der Zuwendung liegen die Werte der Eltern in der vorliegenden Stichprobe verglichen mit vorherigen Studien etwas niedriger [30] bzw. ähnlich hoch [8]. Beide Elternteile geben an, ähnlich „wenig“ belastet zu sein, und scheinen vergleichbar auf die kindlichen chronischen Schmerzen zu reagieren. Ein Grund für die Homogenität der elterlichen Angaben könnte die Erhebung von jeweils beiden Elternteilen eines erkrankten Kindes sein, welche aufgrund eines intensiven Austauschs und langjährigen Zusammenlebens eher ähnliche Einstellungen, Denk- und Verhaltensweisen haben. Ein weiterer Grund könnte – wie auch von Langer et al. [17] beschrieben – die deutliche Belastung der Kinder und der betroffenen Familien aufgrund der kindlichen chronischen Schmerzen in der vorliegenden Stichprobe sein, welche die theoretisch vermuteten Unterschiede überlagert.

Die Kombination von kindlichen Angstsymptomen und schmerzbezogener Beeinträchtigung scheint in der Eltern-Kind-Interaktion eine zentrale Rolle zu spielen und sich verstärkend auf das elterliche Katastrophisieren auszuwirken. Daraus folgt ein dysfunktionaler Teufelskreis: Bei verstärkter kindlicher Angstsymptomatik und zusätzlicher schmerzbezogener Beeinträchtigung des Kindes verstärkt sich das Katastrophisieren der Eltern, was zu mehr zuwendenden Reaktionen führt [19]. Diese wirken sich wiederum negativ auf die kindliche Schmerzsymptomatik aus [4, 5, 14], was bei zusätzlichem Vorliegen von kindlichen Angstsymptomen nach den Ergebnissen der vorliegenden Studie wiederum zu vermehrtem elterlichem Katastrophisieren führt. Zu vermuten ist eine verstärkte Verunsicherung und Belastung des familiären Systems bei Auftreten zweier Symptomcluster beim Kind (schmerzbezogene Beeinträchtigung und Angstsymptome), welche dann zu maladaptiven elterlichen Reaktionen auf die Vermeidungstendenzen des Kindes führt. In Folgestudien sollte daher der oben beschriebene Teufelskreis unter Berücksichtigung der Belastung der Eltern und der Kinder überprüft werden.

Der Alterseffekt der elterlichen Zuwendung erscheint entwicklungspsychologisch nachvollziehbar. Dass Mädchen mehr Zuwendung erhalten als Jungen ist möglicherweise mit ausgeprägterem (faszialem) Schmerzausdruck bei Mädchen zu begründen [12]. Ein weiterer Aspekt könnte sein, dass auf die Schmerzen von Söhnen häufiger mit strafendem/entmutigendem [13] oder ablenkendem Verhalten [17] reagiert wird als auf Schmerzen von Töchtern.

Im Rahmen des Empathiemodells von Goubert et al. [11] lässt sich der auch in vorherigen Studien [30] gezeigte verstärkende Einfluss der elterlichen Angstsymptome als Top-down-Variable auf die Zuwendung erklären. So scheint es weniger die eigene Somatisierung der Eltern als vielmehr die affektive Komponente zu sein, die die elterlichen zuwendenden Reaktionen beeinflusst. Ein zentraler Aspekt von klinischer Angstsymptomatik ist die kognitive Vermeidung von negativ empfundenen Stimuli. Eltern könnten demzufolge versuchen, ihre negativen Gedanken und Emotionen bezüglich der kindlichen Schmerzen durch Zuwendung zu reduzieren.

### Limitationen

Die vorliegende Studie liefert relevante Ergebnisse hinsichtlich der modulierenden Faktoren von maladaptiven elterlichen Reaktionen auf die kindlichen chronischen Schmerzen. Mit der Erhebung von beiden leiblichen Elternteilen und deren schmerzkrankem Kind liegt eine aussagekräftige und bisher weitestgehend einzigartige Stichprobe vor. Des Weiteren wurde in der statistischen Auswertung mit der Anwendung von hierarchischen linearen Modellen die interne Verzerrung der oben genannten Probandentrias berücksichtigt. Nichtsdestotrotz sind einige methodische Einschränkungen zu nennen.

Die Generalisierbarkeit der Ergebnisse ist aufgrund des klinischen Extremsamples von außergewöhnlich hoch belasteten Kindern und Familien eingeschränkt und nicht auf die Allgemeinheit übertragbar. Außerdem wurde in der vorliegenden Studie ausschließlich mit Selbstangaben von Kindern und Eltern gearbeitet und es liegt keine experimentelle Manipulation mit Kontrollgruppe o. Ä. vor. Daher können keine kausalen Aussagen, sondern lediglich Zusammenhänge postuliert werden. Aufgrund der Erhebung in Form von Selbstberichten ist unklar, ob sich die elterliche Selbsteinschätzung tatsächlich im Verhalten widerspiegelt. Hierfür wären kindliche Berichte oder experimentelle Methoden wie „cold pressure tasks“ [2, 21] notwendig.

## Fazit für die Praxis

Zusammenfassend lässt sich feststellen, dass nicht das Geschlecht der Eltern, sondern das gleichzeitige Auftreten von kindlichen Angstsymptomen und schmerzbezogener Beeinträchtigung mit stärkerem elterlichem Katastrophisieren einhergeht. Außerdem verstärken elterliche Angstsymptome die zuwendenden Reaktionen der Eltern. So ist es unabdingbar, bei kindlichen chronischen Schmerzen auch das mögliche zweite Symptomcluster der Angstsymptome mit zu berücksichtigen (und vice versa) sowie Eltern mit Angsterkrankungen hinsichtlich ihrer Reaktionen auf kindliche Schmerzen und Vermeidungstendenzen psychoedukativ zu unterstützen. Ziel sollte sein, den dysfunktionalen Teufelskreis und die darin enthaltenen Zusammenhänge an größeren Stichproben zu überprüfen sowie diesem präventiv und therapeutisch entgegenzuwirken, um somit die Chronifizierung von kindlichem Schmerz zu verringern.
